# Jagged-1 induced molecular alterations in HPV associated invasive squamous cell and adenocarcinoma of the human uterine cervix

**DOI:** 10.1038/s41598-018-27699-1

**Published:** 2018-06-19

**Authors:** Richa Tripathi, Gayatri Rath, Showket Hussain, Poonam Jawanjal, Kapil Bandil, Vishwas Sharma, Mausumi Bharadwaj, Ravi Mehrotra

**Affiliations:** 1 Division of Molecular Genetics & Biochemistry, ICMR-National Institute of Cancer Prevention and Research (NICPR), Noida, India; 2 Division of Preventive Oncology, ICMR-National Institute of Cancer Prevention and Research (NICPR), Noida, India; 3 Department of Anatomy, VMMC & Safdarjung Hospital, New Delhi, India; 4 Department of Health Research, ICMR-National Institute of Cancer Prevention and Research (NICPR), Noida, India; 5 Society for Life Sciences and Human Health, Allahabad, India

## Abstract

The majority of cervical cancer (CC) cases are attributable to HPV infection. Altered Notch pathway signals and HPV are believed to modify clinicopathogenesis of CC, however, the involvement of each molecular player and its mechanism is still not known. Jagged-1 (JAG1) is one of the ligands that induce Notch pathway. The involvement of JAG1 in the modulation of a disease condition is not very clear. Hence, this study aims to study the role of JAG1 in HPV-16/18 associated different histological sub-types of CC, especially ADC. 40 non-neoplastic cervical tissues, 30 precancer and 118 tumor specimens (total 188 tissue biopsies) were studied for the expression of the JAG1 protein through immunohistochemistry, immunoblotting and for HPV infection. Two folds increase of cytoplasmic (Mean ± S.E, 3.67 ± 0.33; p = 0.0001) and nuclear (3.70 ± 0.38, p = 0.0001) JAG1 expression was identified in normal (N) vs precancer and three folds cytoplasmic (4.44 ± 0.17, p = 0.0001) and nuclear (4.64 ± 0.17; p = 0.0001) in N vs. ISCC. Total 85% of ADC patients were found to be infected with HPV, which were 100% infected with HPV-16. These findings suggest the complex synergistic interplay between JAG1 and HPV in regulating clinicopathological progression of CC through its deregulation.

## Introduction

Cervical cancer (CC) ranks 4^th^ in women related malignancies and overall 7^th^ most globally reported carcinoma with high incidence rate (5,28,000 new cases) in 2012^1^. Histologically, CC can be characterized by two different sub-categories i.e. (i) invasive squamous cell carcinomas (ISCC) which are frequent and covers 85–90% of CC cases (ii) adenocarcinomas (ADC) which are relatively rare and comprises only 10–15% cases^2^. The CC develops progression of precancerous lesions; called cervical intraepithelial neoplasia (CIN) grade 1–3 or squamous intraepithelial lesion (SIL)^3^.

Human Papillomavirus (HPV) has emerged as a fundamental regulator of CC^4,5^ and more than 90% of detected infections get cleared within two years^6^. Therefore, the major risk factor of CC is exposure to HPV infection especially HPV-16/18, but to generate and to maintain the malignant processes, HPV infection needs other cofactors; as only few cells infected with carcinogenic HPV develops into CC^7^. Thus, the current thrust area in the field of CC is to illustrate the molecular mechanism/s involved in the initiation and progression of CC, as well as to develop therapeutic targets.

Studies revealed an interaction of HPV proteins with Notch signaling pathway^4^. The Notch is a complex transmembrane pathway involved in a) cell proliferation, differentiation and development^8^, b) organogenesis, c) maintains stem cell viability^9^ and d) renewal in the adult^10^. There are four Notch homologues (Notch 1–4) and five ligands (three Delta-like and two Jagged/Serrate)^11^. Among all ligands, animal studies showed that a null mice for Jagged-1 (JAG1) encoded genes exhibit distinct embryonic defects^12^, suggesting its inimitability independent of canonical Notch pathway. JAG1 binding with Notch-3 dissociates its extracellular unit from the transmembrane unit, sequentially cleaved by proteases, translocating the cleaved intracellular Notch C-terminal fragment to the nucleus and recruits CSL (CBF1, Suppressor of Hairless, Lag1), Mastermind-like proteins (MAML1, 2 and 3) and p300 proteins to transcriptionally regulate target genes.

Till date, there are five studies (Supplement S1) available with respect to JAG1 induced Notch signaling in HPV associated CC. Hence, this study aims to fill the research gap by measuring the expression of JAG1 in various degrees of HPV linked cervical precancer, ISCC and ADC patients by immunohistochemistry, in order to understand its biological role in activation of Notch signaling and to further evaluate its clinical utility in this cancer.

## Results

### Immunohistochemical analysis of JAG1 expression in cervical precancer and cancer

**Figure 1:**
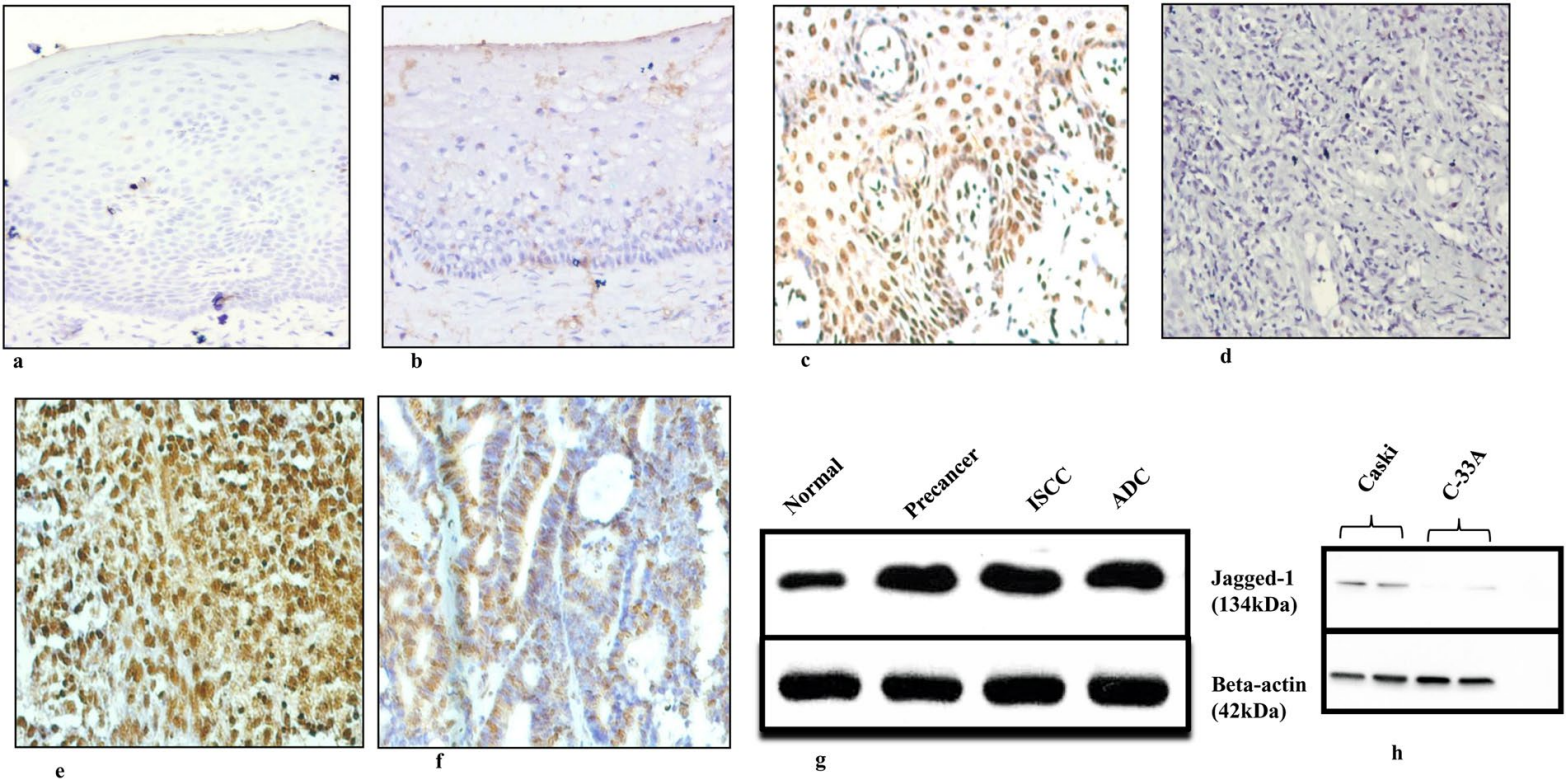
Immunohistochemical analysis of JAG1 in normal, precancer and cancer tissues of uterine cervix. (**a**) negative control in normal tissue, 200X (**b**) mild nuclear and cytoplasmic expression of JAG1 in normal cervix, 200X (**c**) moderate nuclear expression of JAG1 in precancerous tissue, 200X (**d**) negative control in cancer tissue (ISCC, 200X) (**e**) intense cytoplasmic and nuclear localization of JAG1 in ISCC, 200X (**f**) intense nuclear expression of JAG1 in ADC, 200X (**g**) western blots depicting expression pattern of JAG1 protein during the progression of cervical cancer in tissues (normal, precancer, ISCC, ADC) (**h**) western blots showing expression pattern of JAG1 protein in Caski and C-33A cell lines. Protein extracts from cervical tumor biopsies, normal tissues and HPV-16 positive (Caski) and HPV-16 negative (C-33A) cell lines were separated in 10% SDS-PAGE and detected by specific antibody of JAG1. All the blots were stripped and reprobed for β-actin levels to confirm equal loading and the quantitation of bands was performed densitometrically as indicated in materials and methods section.

The expression pattern (Fig. 1a–f) and total expression scores (Table 1) of JAG1 were observed in N, precancer, ISCC and ADC tissues.

**Table 1:** Total expression score (Intensity score + Percentage positivity) of JAG1 expression in Normal, Precancer, ISCC and ADC tissues.

JAG1 (Total expression score)									
Cases	Cytoplasm (C; Mean ± S.E)	p-value*	p-value**	Nucleus (Nu; Mean ± S.E)	p-value*	p-value**	C + Nu (Mean ± S.E)	p-value*	p-value**
Normal (N)	1.10 ± 0.24	N. A	N. A	0.95 ± 0.21	N. A	N. A	1.03 ± 0.19	N. A	N. A
Precancer	3.67 ± 0.33	N. A	N. A	3.70 ± 0.38	N. A	N. A	3.68 ± 0.33	N. A	N. A
ISCC	4.44 ± 0.17	N. A	N. A	4.64 ± 0.17	N. A	N. A	4.56 ± 0.16	N. A	N. A
ADC	3.05 ± 0.40	N. A	N. A	4.20 ± 0.40	N. A	N. A	3.63 ± 0.23	N. A	N. A
N vs Precancer	N. A	**0**.**0001**	**0**.**0001**	N. A	**0**.**0001**	**0**.**0001**	N. A	**0**.**0001**	**0**.**0001**
N vs ISCC	N. A	**0**.**0001**	**0**.**0001**	N. A	**0**.**0001**	**0**.**0001**	N. A	**0**.**0001**	**0**.**0001**
N vs ADC	N. A	**0**.**0001**	**0**.**0001**	N. A	**0**.**0001**	**0**.**0001**	N. A	**0**.**0001**	**0**.**0001**
Precancer vs ISCC	N. A	0.3080	**0**.**0230**	N. A	0.0830	**0**.**0190**	N. A	0.3640	**0**.**0110**
ADC vs ISCC	N. A	0.2920	**0**.**0010**	N. A	0.8360	0.1600	N. A	0.2980	**0**.**0020**

*p-values were calculated using Student’s t-test for comparing the means between normal vs precancer, normal vs ISCC, normal vs ADC; precancer vs ISCC and ADC vs ISCC.

**p-values were calculated using chi-square test after taking cutoff values of respective total scores. Abbreviations: ADC, Adenocarcinoma; ISCC, Invasive squamous cell carcinoma; N.A, not applicable.

#### Cytoplasm (C)

Two folds (Mean ± S.E, 3.67 ± 0.33; p = 0.0001) increase of cytoplasmic JAG1 expression was identified in N vs. precancer, three folds (4.44 ± 0.17; p = 0.0001) in N vs. ISCC. However, only two folds (3.05 ± 0.40; p = 0.0001) increase was found in N vs. ADC.

#### Nucleus (Nu)

JAG1 expression in the nucleus was found to be increased two folds (Mean ± S.E, 3.70 ± 0.38; p = 0.0001) in N vs. precancer, three folds (4.64 ± 0.17; p = 0.0001) in N vs. ISCC and N vs. ADC (4.20 ± 0.40; p = 0.0001).

#### C + Nu

In accordance, two folds (3.68 ± 0.33; p = 0.0001) increase was observed in N vs. precancer and three folds (4.56 ± 0.16; p = 0.0001) elevation in N vs. ISCC. Only two folds (3.63 ± 0.23; p = 0.0001) increase was observed in N vs. ADC.

**Figure 2:**
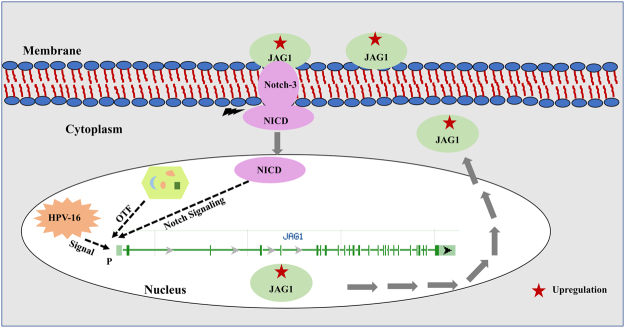
JAG1 induced deregulated Notch signaling in HPV associated cervical carcinogenesis. Red arrow shows upregulation and green arrow shows downregulation. Abbreviations: NICD, notch intracellular domain; OTF, other transcription factors; P, Promoter.

Hence, we hypothesize that JAG1, while binding to Notch-3, gives a signal which results in the cleavage of NICD domain (Fig. 2). The cleaved part translocates to the nucleus where it gives activating signal to transcription factor/s (TFs) of *JAG1* gene. Moreover, HPV-16 gives an additional signal to the TFs of *JAG1* gene (based on other experimental results on HPV given below) which cumulatively activates the transcription of *JAG1* and further results to the increase production of JAG1 protein in the nucleus. The JAG1 protein once formed translocate to the cytoplasm, hence, increasing its level in the cytoplasm.

### Evaluation of JAG1 potential to distinguish precancer, invasive squamous cell carcinoma and adenocarcinoma from normal cervix tissue

Receiver Operating Characteristic (ROC) analysis was done to estimate the JAG1 potential as a diagnostic marker for all three groups (precancer, ISCC and ADC) of the uterine cervix.

#### Cytoplasm

The values for AUC (area-under-the-curve) for JAG1 in precancer (0.86; p = 0.01), ISCC (0.91; p = 0.0001) and ADC (0.81; p = 0.0001) was found to be significant.

#### Nucleus

**Figure 3:**
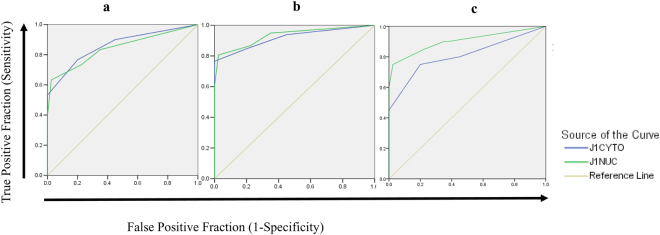
(**a–c**) Receiver operating characteristic curves of JAG1 nuclear and cytoplasmic in (**a**) Normal vs. Precancer, (**b**) Normal vs. ISCC and (**c**) Normal vs. ADC. The blue line shows ROC analysis for cytoplasmic JAG1. The green line shows ROC analysis for nuclear JAG1 respectively. Y-axis of the plot shows true-positive fraction and X-axis shows the false positive fraction.

Similarly, the significant AUC values for nuclear JAG1 in precancer (0.84; p = 0.0001), ISCC (0.93; p = 0.0001) and ADC (0.90; p = 0.0001) were observed. The sensitivity and specificity for cytoplasmic JAG1 were 76.7%, 84.7%, 75%; and 80%, 80%, 80% respectively. In accordance, for nuclear JAG1 these were 73.3%, 86.7%, 85% and 77.5, 77.5%, 80% respectively {Fig. 3(a–c) and Table S1}.

High sensitivity and specificity of JAG1 in precancer, ISCC and ADC support the clinical utility of JAG1 for early detection and progression of cervical cancer.

### Relation of immunohistochemical expression of JAG1 with clinicopathological parameters of ISCC

An association of JAG1 expression with clinicopathological parameters of ISCC has been described as follows (Table 2).

**Table 2:** Analysis of JAG1 protein expression in ISCC and its correlation with demographic and clinicopathological parameters.

Parameters	Total JAG1(Cyto)				JAG1(Nuclear)				JAG1(Cyto + Nucl)			
	N	− (%)	+ (%)	p-value	N	− (%)	+ (%)	p-value	N	− (%)	+ (%)	p-value
Age < 50	15	5(33.3)	10(66.7)	0.73	13	4(30.8)	9(69.2)	0.92	13	5(38.5)	8(61.6)	0.45
Age ≥ 50	83	24(28.9)	59(71.1)		85	25(29.4)	60(70.6)		85	24(28.2)	61(71.8)	
Gravidity < 3	16	1(6.3)	15(93.8)	0.27	—	1(6.3)	15(93.8)	0.36	—	1(6.3)	15(93.8)	0.36
Gravidity ≥ 3	82	14(17.1)	68(82.9)		—	12(14.6)	70(85.4)		—	12(14.6)	70(85.4)	
Parity < 3	18	4(22.2)	14(77.8)	0.36	—	4(22.2)	14(77.8)	0.21	—	3(16.7)	15(83.3)	0.63
Parity ≥ 3	80	11(13.8)	69(86.3)		—	9(11.3)	7(88.8)			10(12.5)	70(87.5)	
No Smoking	63	12(19)	51(81)	0.16	—	9(14.3)	54(85.7)	0.68	—	10(15.9)	53(84.1)	0.30
Smoking	35	3(8.6)	32(91.4)		—	4(11.4)	31(88.6)		—	3(8.6)	32(91.4)	
No tobacco	62	10(16.1)	52(83.9)	0.76	—	9(14.5)	53(85.5)	0.63	—	8(12.9)	54(87.1)	0.89
Tobacco	36	5(13.9)	31(86.1)		—	4(11.1)	32(88.9)		—	5(13.9)	31(86.1)	
Tumor size < 4	16	4(25)	12(75)	0.23	—	4(25)	12(75)	0.13	—	4(25)	12(75)	0.13
Tumor size ≥ 4	82	11(13.4)	71(86.6)		—	09(11)	73(89)		—	9(11)	73(89)	
No vaginal invo	33	10(30.3)	23(69.7)	*0.003	—	9(27.3)	24(72.7)	*0.004	—	9(27.3)	24(72.7)	*0.001
Vaginal invo	65	5(7.7)	60(92.3)		—	4(6.2)	61(93.8)		—	4(62)	61(93.8)	
Grade G1	46	10(21.7)	36(78.3)	0.09	—	8(17.4)	38(82.6)	0.25	—	8(17.4)	38(82.6)	0.25
Grade G2 + G3	52	5(9.6)	47(90.4)			5(9.6)	47(90.4)			5(9.6)	47(90.4)	
No lymph Nodes	33	9(27.3)	24(72.7)	*0.01	—	7(21.2)	26(78.8)	*0.05	—	8(24.2)	25(75.8)	*0.02
Lymph Nodes	65	6(9.2)	59(90.8)		—	6(9.2)	59(90.8)		—	5(7.7)	60(92.3)	
Figo Stage I + II	33	10(30.3)	23(69.7)	*0.003	—	9(27.3)	24(72.7)	*0.004	—	9(27.3)	24(62.7)	*0.004
lII + IV	65	5(7.7)	60(92.3)		—	4(6.2)	61(93.8)		—	4(6.2)	61(93.8)	

^*^p ≤ 0.05 is considered as significant.

Abbreviations: ISCC, Invasive squamous cell carcinoma; N, number of subjects.

#### Cytoplasm

JAG1 overexpression showed an association with lymph nodes metastasis (90.8%; p = 0.01), vaginal involvement of tumor (92.3%; p = 0.003), as well as with Figo stage (92.3%; p = 0.003) respectively.

#### Nucleus

Tumor vaginal involvement (93.8%; p = 0.004), lymph nodes metastasis (90.8%; p = 0.05) and Figo stage (93.8%; p = 0.004) were found to be associated with JAG1 expression.

#### C + Nu

Similarly, JAG1 was associated with tumor vaginal involvement (93.8%; p = 0.001), lymph nodes metastasis (92.3%; p = 0.02) as well as with Figo stage (93.8%; p = 0.004) respectively.

The results imply the association of JAG1 with the aggressive behavior of the CC.

### Immunoblotting

A gradual increase in the expression of JAG1 (134 kDa) was identified in precancer, ISCC, and ADC patient samples (Fig. 1g, S1a,b). Immunoblotting results were also validated in Caski and C-33A cell lines. Caski showed increased expression as compared to C-33A (Fig. 1h, S2). The above results validate the findings of immunohistochemistry.

### Prevalence of HPV infection in ADC and its correlation with clinicopathological parameters

Previously, we identified the prevalence of HPV infection in precancer and ISCC tissues^13^. In this study, 85% (17/20) of ADC patients were identified to be infected with HPV. Among them, all the patients were found to be infected with HPV-16 (17/17) and 5.8% (01/17) were found to be co-infected with HPV-18. All the ADC patients had 100% Grade II + III and Figo stage III + IV, and hence all HPV infected ADC were of higher pathological grades.

Hence, clinicopathological progression of ADC confirms the involvement of HPV-16.

### Association of HPV-16/18 infection and Jagged-1 in precancer, ISCC, and ADC

Association of HPV with JAG1 expression in all tissues is defined as follows (Table 3):

**Table 3:** Correlation of HPV infection with JAG1 expression in Precancer, ISCC and ADC.

HPV types	Status	Total cases	JAG1 cyto −n(%) + n(%)	p-value	JAG1 nuclear − n(%) + n(%)	p-value	JAG1 (C + N) n(%) n(%)	p-value
**Precancer (n = 30)**								
HPV-16	No	06	5(83.3) 1(16.7)	*0.0001	5(83.3) 1(16.7)	*0.0002	4(66.7) 2(33.3)	*0.007
	Yes	24	2(8.3) 22(91.7)		3(12.5) 21(87.5)		2(8.3) 22(91.7)	
HPV-18	No	30	7(23.3) 23(76.7)	—	7(23.3) 23(76.7)	—	7(23.3) 23(76.7)	—
	Yes	00	0(0) 0(0)		0(0) 0(0)		0(0)0(0)	
HPV overall positive	No	06	5(83.3) 1(16.7)	*0.0001	5(83.3) 1(16.7)	*0.0002	4(66.7) 2(33.3)	*0.007
	Yes	24	2(8.3) 22(91.7)		2(8.3) 22(91.7)		2(8.3) 22(91.7)	
**ISCC (n = 98)**								
HPV-16	No	16	10(62.5) 6(37.5)	*0.0001	08(50) 08(50)	*0.0001	8(50) 08(50)	*0.0001
	Yes	82	5(6.1) 77(94)		5(6.1) 77(94)		5(6.1) 77(93.9)	
HPV-18	No	92	14(15.2) 78(85)	0.924	13(14.1) 79(86)	0.323	12(13) 80(87)	0.800
	Yes	06	1(16.7) 05(83.3)		0(0) 6(100)		1(16.7) 5(83.3)	
HPV Overall positive	No	13	9(69.2) 4(30.8)	*0.0001	8(61.5) 5(38.5)	*0.0001	7(53.3) 6(46.2)	*0.0001
	Yes	85	6(7.1) 79(92.9)		5(5.9) 80(94.1)		6(46.2) 79(92.9)	
**ADC (n = 20)**								
HPV-16	No	03	0(0) 03(100)	0.39	0(0) 03(100)	0.59	0(0) 03(100)	0.85
	Yes	17	5(29.4) 12(70.6)		3(17.6) 14(82.4)		1(5.9) 16(94.1)	
HPV-18	No	19	4(21.1) 15(78.9)	0.39	3(15.8) 16(84.2)	0.85	1(5.3) 18(94.7)	0.95
	Yes	01	1(100) 0(0)		0(0) 1(100)		0(0) 01(100)	
HPV Overall positive	No	03	0(0) 03(100)	0.39	0(0) 03(100)	0.59	0(0) 03(100)	0.90
	Yes	17	5(29.4) 12(70.6)		3(17.6) 14(82.4)		1(5.9) 16(94.1)	

^*^p ≤ 0.05 is considered as significant.

#### Precancer

HPV-16 positive precancer patients showed 91.7% (p = 0.0001) cytoplasmic, 87.5% (p = 0.0002) nuclear and 91.7% (p = 0.0007) cytoplasmic + nuclear JAG1 positivity (Table 3). None of the precancer patients were found to be infected with HPV-18. Among precancer patients, HPV-16 positive CIN-1 and CIN-2/3 cases showed 84.2%, 78.9%; 63.6%, 63.6% JAG1 cytoplasmic and nuclear positivity respectively (Table S2).

#### ISCC

HPV-16 positive ISCC patients showed 94% nuclear and 94% cytoplasmic JAG1 positivity. This showed that in ISCC patients, nuclear (p = 0.0001) and cytoplasmic (p = 0.0001) JAG1 were observed to be correlated significantly with HPV-16.

#### ADC

No significant association of JAG1 with HPV-16 positive ADC patients was observed.

#### Cell lines

Increased expression of JAG1 was depicted in Caski (HPV-16 positive) as compared to C-33A (HPV-16 negative) cell lines.

This strengthens the hypothesis that HPV-16 associated precancer and cervical tumorigenesis is synergized with altered Notch signaling.

### Associations between JAG1 and Notch-3 Protein in Precancer and ISCC patients

Interprotein correlations was analyzed between Notch-3 and JAG1 proteins. In precancer patients, significant positive associations were found between JAG1 nuclear (r = 0.530, p = 0.003) and Notch-3 nuclear proteins (Table S3). Similarly, in ISCC patients significant positive associations were observed between JAG1 nuclear (r = 0.379, p = 0.0001), cytoplasmic (r = 0.479, p = 0.0001) and cytoplasmic + nuclear (r = 0.453, p = 0.0001) and Notch-3 proteins (Table S4).

## Discussion

The development and advancement of tumor from precancer to ISCC and ADC of uterine cervix remains a major clinical problem for decades. Evidence suggests that HPV infection alone is inadequate to induce malignant changes^14^. The network of altered signaling pathways are also important for the development of CC^13^. Therefore, for defining an effective treatment strategy, it is essential to understand the molecular mechanism/s involved in CC cells infected with oncogenic HPV types. Knowledge of this may improve the diagnostic modalities for precancer and cancer patients of uterine cervix and defining treatment strategies. This study is the first step towards this direction, where the role of JAG1 in the etio-pathogenesis of HPV infected different histological sub-types of CC is defined. Along with ISCC, we have also included a very rare form of CC i.e. ADC in our study. The outcome of this study will aid clinicians in deciding the targeted therapeutic strategy for CC patients and researchers in understanding the involvement of JAG1 in the pathogenesis of CC.

The results of JAG1 protein expression profile through immunohistochemistry suggest the JAG1 binding with Notch-3 triggers Notch signaling in HPV associated CC, which in-turn activates TFs for *JAG1* expression. The JAG1 protein produced in the nucleus accumulates and translocates from nucleus to cytoplasm. Our JAG1 expression results demonstrate altered expression levels of JAG1 protein which results in deregulation of the Notch pathway. This overall may support the infection of the cervical mucosa by HPV-16. Together, this may lead to the acceleration of the cell cycle with an acquisition of more genetic damage. Our results are in agreement with a study^15^ who showed an intense immunoreactivity of JAG1. However, this study elaborated and showed that cytoplasmic and nuclear expression of JAG1 in HPV associated precancer, ISCC and ADC cases. Based on cytoplasmic and nuclear expression pattern we have explained the probable mechanism of the JAG1 feedback loop.

The ROC curve analysis of JAG1 showed its high sensitivity and specificity suggesting its clinical utility in early detection and progression of CC patients.

Immunohistochemical expression of JAG1, with respect to clinicopathological parameters, showed its association with the tumor vaginal involvement, Figo stage and lymph node metastasis, highlighting its clinical utility in ISCC. The upregulated JAG1 expression may interact with aggressive tumor behaviour, tumor progression and expansion, predicting as a potential candidate for biomarker of disease progression. This supports the findings of Yousif *et al*.^16^ who also identified the correlation of JAG1 expression levels in tumor patients with variable tumor clinicopathological parameters but through western blotting.

The IHC results corroborated well with immunoblotting and showed concordance with Yousif *et al*.^16^ who identified increased JAG1 levels in CC tissues by real- time PCR and western blotting. This study expanded with respect to HPV associated precancer, ISCC and ADC patients by IHC as well as Immunoblotting.

We studied earlier the prevalence of HPV infection in precancer and in invasive CC biopsies, and observed the linkage of HPV positive cancers with clinicopathological parameters of the disease progression^13^. This indicates that E6 and E7 oncogenic proteins of HPV can interact with the activated JAG1 which in-turn activate various pathways associated in cancers and altogether synergizes with each other, inhibiting apoptosis, promoting cell proliferation and tumorigenesis^17^. This study did not find any significant association of JAG1 with HPV-16 positive ADC subjects which could be due to the limited number of ADC samples (n = 20) being analyzed.

We validated the increased expression of JAG1 in Caski, as compared to C-33A cell lines, invasive cervical tumor-derived cell lines. Similar expression pattern of JAG1 was also previously reported in Caski by Veeraraghavalu *et al*.^18,19^.

Human studies and *in vitro* cell line experiments strongly support the hypothesis that JAG1 is the major ligand in triggering activation of Notch signaling, which acts as a central player in HPV-16 associated CC development and progression. Therefore, cancer cells expressing JAG1 plays pivotal roles in two manners: (i) JAG1 acts as a ligand and complexed with its receptor activating neighboring cells of a tumor in a juxtacrine way. (ii) The ICD (intracellular domain) of JAG1 may initiate Notch pathway and propagate tumor cell growth, leading to deregulation of JAG1. This creates a favorable environment for the growth of early precancerous lesions, acting as a protagonist in the tumor development and proliferation. Hence, serves a probable transducer of this cancer.

A better understanding of crosstalk between Notch signaling and other pathways, such as Wnt, Hedgehog, and vascular endothelial growth factor (VEGF), as well as with the immune system can provide valuable clues how to target cancer survival by ablating them and will make JAG1, an attractive approach orchestrated for combination therapy. Furthermore, due to its anti-apoptotic and pro-“stemness” functions, JAG1 blockade epitomizes an alluring model for combination therapy orchestrated by standard chemotherapy as demonstrated in preclinical models of ovarian and pancreatic cancer and lymphoma^20,21^.

In conclusion, our findings excavate the understanding of JAG1 driven Notch signaling events in HPV associated progression of CC. JAG1 expression in the nucleus denotes the transcription of *JAG1* gene due to NICD and HPV-16 signaling to TFs resulting in JAG1 protein in nucleus. However, JAG1 expression in cytoplasm clarifies its translocation from nucleus to cytoplasm which in-turn activates the Notch signaling via binding to Notch-3, hence, acting as a positive feedback loop. Thus, it provides a legitimate target for CC therapy in the hope it might lead to the development of such tailored individualized therapy, as well as characterizing its biological functions in the cells which may be sufficient to abolish the neoplastic phenotype. This may help clinicians in characterizing patients when selecting for treatment of such cancer, by developing chemotherapeutic and combination therapies to interfere with cancer invasion and metastasis. Further validations at an RNA transcript level by RNA Seq can be done in future studies. In addition, studies are desired to assess JAG1 ablation as an adjuvant treatment to existing and currently ineffective chemotherapeutic agents.

## Methods

### Study design and participants

**Figure 4:**
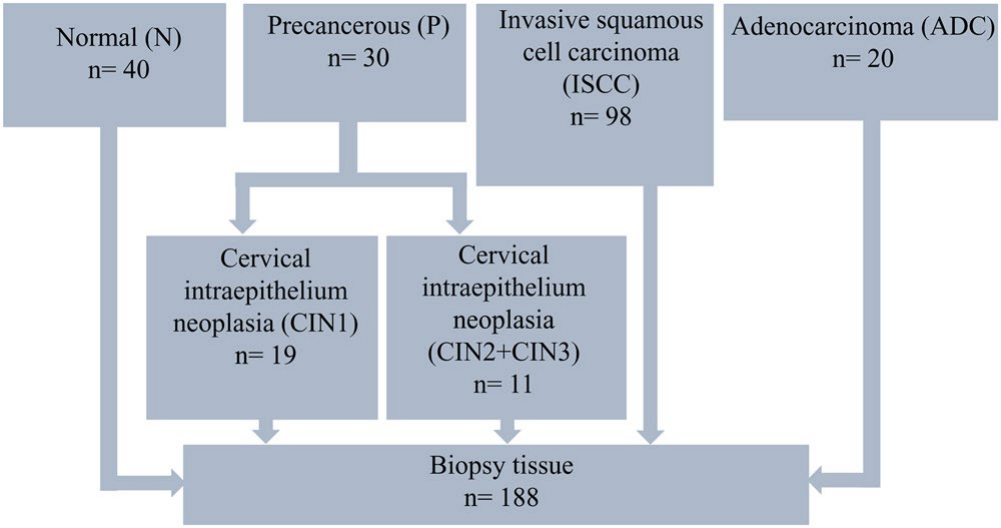
Study design.

118 tumor specimens (98 ISCC, 20 ADC), 30 precancer and 40 non-neoplastic cervical tissues (total 188 tissue biopsies, Fig. 4) were collected after taking the written informed consent from all participating patients during the clinical procedures at Department of Obstetrics and Gynecology, Safdarjang Hospital, New Delhi, India. All patients in the study were recruited having no prior treatment and no family history of any disease or CC. This study was approved by the institutional ethics committee of ICMR-National Institute of Cancer Prevention and Research, Noida, as well as from Vardhman Mahavir Medical College and Safdarjang Hospital, New Delhi. Also, the study confirms that all experiments were performed in accordance with relevant guidelines and regulations.

The clinicopathological characteristics of patients with CC were recorded according to previous studies^13,22^. The staging of the tumors was carried out according to the criterion of the International Federation of Gynecology and Obstetrics (FIGO) classification of tumor staging^23^. Each slide was evaluated for its respective histopathological grade and clinical staging by two independent pathologists^22,23^. Each collected sample was distributed into two parts, one for histopathological diagnosis and other stored at −70 °C to perform molecular analysis.

### IHC

All the collected specimens were fixed with 10% formalin, embedded in paraffin and then cut in 5μm sections, further mounted on priorly coated poly-L-lysine (Sigma, St. Louis, MO, USA) slides. Conventional H&E staining was done in each section followed by immunohistochemistry. Tris EDTA (pH 9.0) was used for antigen retrieval in the microwave and incubated overnight at 4^0^C with the primary rabbit polyclonal antibody of JAG-1 (ab7771 Abcam) at a dilution of 1:200. After washing with tris buffer saline (TBS), pH 7.4, sections were incubated consequently with polymer based Envision™ (An Envision System peroxidase kit, DAKO, Carpinteria, CA) as described by us previously^13^. The images were captured using Olympus microscope (model BX-51, Olympus America, Inc., Melville, NY) using Olympus Bio-report software. 3,3-diaminobenzidine hydrochloride (DAB) was used for color development followed by its counter staining by Mayer’s Hematoxylin.

### IHC Evaluation

The slides of IHC were independently reviewed by two authors along with one histopathologist independently without prior knowledge of patient’s identity, and were further scored as described previously^13^. Percentage positivity score was then combined with intensity scores in order to get the final score.

### Immunoblotting

Immunoblotting was performed in representative cases from all categories (normal, precancer, ISCC and ADC) of collected biopsy tissues and cell lines according to our previous studies^3,13,24^. The primary antibodies used were rabbit polyclonal antibody of JAG-1 (ab7771 Abcam, 1:1000), and rabbit monoclonal β-actin (1:2000, Abcam, US). Blots were developed using enhanced chemiluminescence ECL detection system (Santa Cruz Biotech, USA). The quantitation and band intensity comparison of JAG1 was done between the subset of normal, precancer and cancer tissues, as well as between C-33A and Caski cell lines respectively by densitometry as mentioned previously^3,24^. Alpha Digidoc version 4.1.0 (Alpha Innotech Corporation, IL) was used for the evaluation of expression.

### Cell culture

Human CC cell lines HPV-16 positive (Caski) and HPV-16 negative (C-33A) were purchased from NCCS, Pune (http://www.nccs.res.in/) in December 2017 and January 2018 respectively. These cell lines were tested and authenticated directly from NCCS, Pune from where they were purchased. Sixteen short tandem repeat (STR) loci were amplified using commercially available AmpFISTR® Identifier® Plus PCR amplification kit from Applied Biosystems. The cell line samples were processed using the Applied Biosystems® 3500 Genetic Analyser. Data was analyzed using Gene Mapper® ID-X v1.5 software (Applied Biosystems) along with appropriate positive and negative controls. The cells were last tested at the time of their purchase. They were culture in DMEM supplemented with 5% FBS, 100 U/mL penicillin, and 100 µg/mL streptomycin at 37 °C in an atmosphere of 5% CO_2_.

### Genomic DNA isolation, and PCR detection of HPV isotypes

Genomic DNA was extracted from the ADC samples following phenol-chloroform method^24,25^. Duplex PCR assay was performed using the consensus sequence primers, for a conserved L1 gene of HPV genome, and by primers specifically designed for HPV-16 and HPV-18. The detailed protocol was mentioned earlier^3^.

### Statistics

SPSS (Version 20) software package was used to perform statistical analysis. Chi-square test was used to determine the expression of the JAG1 protein with the clinicopathological parameters of cancer patients. Interprotein correlations was observed between Notch-3 and JAG1 proteins by Spearman’s rank correlation test. In doing so, the data of Notch-3 was obtained from our previous published report^13^. The p-value ≤ 0.05 was considered as statistically significant.

### Data availability

The datasets generated during and/or analyzed during the current study are available from the corresponding author on reasonable request.

### Summary

This study describes the role of Jagged-1 in context to HPV infection, in the pathogenesis of different histological subtypes of cervical carcinoma. It provides the molecular circuitry regulating Notch signaling and a novel therapeutic opportunity of JAG1 ablation, aiding clinicians.

## Electronic supplementary material

Supplement S1, Table-S1, Table-S2,Table-S3, Table-S4, Figure S1, Figure S2
