# Supplementary material for: Jagged-1 induced molecular alterations in HPV associated invasive squamous cell and adenocarcinoma of the human uterine cervix
Source: Sci Rep. 2018 Jun 19;8:9359. doi: 10.1038/s41598-018-27699-1 (PMC6008329; doi:10.1038/s41598-018-27699-1)

Jagged-1 induced molecular alterations in HPV associated invasive squamous cell and adenocarcinoma of human uterine cervix

Richa Tripathi, PhD; Gayatri Rath, MS; Showket Hussain, PhD; Poonam Jawanjal PhD; Kapil Bandil, M.Tech; Vishwas Sharma, PhD; Mausumi Bharadwaj, PhD; Ravi Mehrotra, MD

Supplement S1

Criteria of screening studies

The screening of the studies was performed on PubMed on 4th of January, 2018. A total of 22 studies were obtained during the comprehensive literature search, of which i) 15 were not related to JAG1,[1–15] ii) 4 were reviews[16–19] iii) 3 were relevant to our interest,[20–22] of which 2[20,21] were solely based on *in vitro* experiments and 1 was our previous report of Notch receptors on CC[22].

In addition, Google was also consulted on 4th of January 2018, so as to incorporate studies not captured through PubMed. Through this 2 additional studies were trapped[23,24]. Both have not described CC with respect to HPV status and have also not classified its sub-types ADC and ISCC.

The details of phrases used are given below:

1. Phrase in PubMed

(("jagged-1 protein"[MeSH Terms] OR ("jagged-1"[All Fields] AND "protein"[All Fields]) OR "jagged-1 protein"[All Fields] OR "jagged 1"[All Fields]) OR JAG1[All Fields]) AND Notch[All Fields] AND (HPV[All Fields] OR ("papillomaviridae"[MeSH Terms] OR "papillomaviridae"[All Fields] OR ("human"[All Fields] AND "papilloma"[All Fields] AND "virus"[All Fields]) OR "human papilloma virus"[All Fields])) AND ("uterine cervical neoplasms"[MeSH Terms] OR ("uterine"[All Fields] AND "cervical"[All Fields] AND

"neoplasms"[All Fields]) OR "uterine cervical neoplasms"[All Fields] OR ("cervical"[All Fields] AND "cancer"[All Fields]) OR "cervical cancer"[All Fields])

2. Phrase in Google

“Jagged-1 AND cervical cancer”.

References

1. Wang, J. & Yue, X. Role and importance of the expression of transcription factor FOXC2 in cervical cancer. *Oncol. Lett.* **14,** 6627–6631 (2017).

2. Kranjec, C. *et al.* Modulation of basal cell fate during productive and transforming HPV-16 infection is mediated by progressive E6-driven depletion of Notch. *J. Pathol.* **242,** 448–462 (2017).

3. Padash Barmchi, M. *et al.* A Drosophila Model of HPV E6-Induced Malignancy Reveals Essential Roles for Magi and the Insulin Receptor. *PLoS Pathog.* **12,** e1005789 (2016).

4. Feng, S., Yang, Y., Lv, J., Sun, L. & Liu, M. Valproic acid exhibits different cell growth arrest effect in three HPV-positive/negative cervical cancer cells and possibly via inducing Notch1 cleavage and E6 downregulation. *Int. J. Oncol.* **49,** 422–30 (2016).

5. Koh, L. F., Ng, B. K., Bertrand, J. & Thierry, F. Transcriptional control of late differentiation in human keratinocytes by TAp63 and Notch. *Exp. Dermatol.* **24,** 754–60 (2015).

6. Halim, T. A., Farooqi, A. A. & Zaman, F. Nip the HPV encoded evil in the cancer bud: HPV reshapes TRAILs and signaling landscapes. *Cancer Cell Int.* **13,** 61 (2013).

7. Henken, F. E. *et al.* The functional role of Notch signaling in HPV-mediated

transformation is dose-dependent and linked to AP-1 alterations. *Cell. Oncol. (Dordr).* **35,** 77–84 (2012).

8. Kuncharin, Y., Sangphech, N., Kueanjinda, P., Bhattarakosol, P. & Palaga, T. MAML1 regulates cell viability via the NF-κB pathway in cervical cancer cell lines. *Exp. Cell Res.* **317,** 1830–40 (2011).

9. Santos, L. *et al.* Identification of differential expressed transcripts in cervical cancer of Mexican patients. *Tumour Biol.* **32,** 561–8 (2011).

10. Talora, C. *et al.* Constitutively active Notch1 induces growth arrest of HPV-positive cervical cancer cells via separate signaling pathways. *Exp. Cell Res.* **305,** 343–54 (2005).

11. Chakrabarti, O. *et al.* Human papillomavirus type 16 E6 amino acid 83 variants enhance E6-mediated MAPK signaling and differentially regulate tumorigenesis by notch signaling and oncogenic Ras. *J. Virol.* **78,** 5934–45 (2004).

12. Thorland, E. C., Myers, S. L., Gostout, B. S. & Smith, D. I. Common fragile sites are preferential targets for HPV16 integrations in cervical tumors. *Oncogene* **22,** 1225–37 (2003).

13. Weijzen, S., Zlobin, A., Braid, M., Miele, L. & Kast, W. M. HPV16 E6 and E7 oncoproteins regulate Notch-1 expression and cooperate to induce transformation. *J. Cell. Physiol.* **194,** 356–62 (2003).

14. Talora, C., Sgroi, D. C., Crum, C. P. & Dotto, G. P. Specific down-modulation of Notch1 signaling in cervical cancer cells is required for sustained HPV-E6/E7 expression and late steps of malignant transformation. *Genes Dev.* **16,** 2252–63 (2002).

15. Tewari, K. S. *et al.* Development and assessment of a general theory of cervical carcinogenesis utilizing a severe combined immunodeficiency murine-human xenograft model. *Gynecol. Oncol.* **77,** 137–48 (2000).

16. Jayshree, R. S., Sreenivas, A., Tessy, M. & Krishna, S. Cell intrinsic & extrinsic factors in cervical carcinogenesis. *Indian J. Med. Res.* **130,** 286–95 (2009).

17. Vande Pol, S. B. & Klingelhutz, A. J. Papillomavirus E6 oncoproteins. *Virology* **445,** 115–37 (2013).

18. Chen, J. Signaling pathways in HPV-associated cancers and therapeutic implications. *Rev. Med. Virol.* **25 Suppl 1,** 24–53 (2015).

19. Wang, F., Li, B. & Xie, X. The roles and clinical significance of microRNAs in cervical cancer. *Histol. Histopathol.* **31,** 131–9 (2016).

20. Veeraraghavalu, K. *et al.* Complementation of human papillomavirus type 16 E6 and E7 by Jagged1-specific Notch1-phosphatidylinositol 3-kinase signaling involves pleiotropic oncogenic functions independent of CBF1;Su(H);Lag-1 activation. *J. Virol.* **79,** 7889–98 (2005).

21. Veeraraghavalu, K. *et al.* Papillomavirus-mediated neoplastic progression is associated with reciprocal changes in JAGGED1 and manic fringe expression linked to notch activation. *J. Virol.* **78,** 8687–700 (2004).

22. Tripathi, R. *et al.* Clinical impact of de-regulated Notch-1 and Notch-3 in the development and progression of HPV-associated different histological subtypes of precancerous and cancerous lesions of human uterine cervix. *PLoS One* **9,** e98642 (2014).

23. Yeasmin, S. *et al.* Expression of nuclear Notch3 in cervical squamous cell carcinomas and its association with adverse clinical outcomes. *Gynecol. Oncol.* **117,** 409–16 (2010).

24. Yousif, N. G. *et al.* Notch1 ligand signaling pathway activated in cervical cancer: poor prognosis with high-level JAG1/Notch1. *Arch. Gynecol. Obstet.* **292,** 899–904 (2015).

Table S1 Test Performance of Jagged-1 (nuclear & cyto) protein in precancer, ISCC and ADC

	Cells	Cut-off value (Total score)	Sensitivity (%)	Specificity (%)	AUC	p-value
**Precancer**	nuclear	2.5	73.3	77.5	0.84	***0.0001**
	cyto	2.5	76.7	80	0.86	***0.01**
**ISCC**	nuclear	2.5	86.7	77.5	0.93	***0.0001**
	cyto	2.5	84.7	80	0.91	***0.0001**
**ADC**	nuclear	2.5	85	80	0.90	***0.0001**
	cyto	2.5	75	80	0.81	***0.0001**

Abbreviation: AUC: Area under curve

$p \leq 0.05$ is considered as significant

Table S2 Correlation of HPV-16 infection with Jagged-1 protein expression in CIN-1 and CIN2/3

	Total cases	Jagged-1 cyto		p-value	Jagged-1 nuclear		p-value
		- n(%)	+ n(%)		- n(%)	+ n(%)	
**HPV-16 in CIN-1**	19	3(15.8)	16(84.2)	0.19	4(21.1)	15(78.9)	0.11
**HPV-16 inCIN-2/3**	11	4(36.4)	7(63.6)	0.19	4(36.4)	7(63.6)	0.11

$p \leq 0.05$ is considered as significant

Table S3 Associations between JAG1 and Notch-3 protein in Precancer patients

Proteins Expression			Notch-3			JAG1		
			C	N	C+N	C	N	C+N
**Jagged-1**	**C**	**R**	0.148	**0.425**	0.298	1.00	**0.714**	**0.905**
		**p value**	0.434	**0.019**	0.110	-	**0.0001**	**0.0001**
	**N**	**R**	**0.444**	**0.530**	**0.526**	**0.714**	1.000	**0.936**
		**p value**	**0.014**	**0.003**	**0.003**	**0.0001**	-	**0.0001**
	**C+N**	**R**	0.323	**0.508**	**0.441**	**0.905**	**0.936**	1.000
		**p value**	0.082	**0.004**	**0.015**	**0.0001**	**0.0001**	-
**Notch-3**	**C**	**R**	1.000	**0.616**	**0.869**	0.148	**0.444**	0.323
		**p value**	-	**0.0001**	**0.0001**	0.434	**0.014**	0.082
	**N**	**R**	**0.616**	1.00	**0.893**	**0.425**	**0.530**	**0.508**
		**p value**	**0.0001**	-	**0.0001**	**0.019**	**0.003**	**0.004**
	**C+N**	**R**	**0.869**	**0.869**	1.00	0.298	**0.526**	**0.441**
		**p value**	**0.0001**	**0.0001**	-	0.110	**0.003**	**0.015**

Abbreviations: C= cytoplasmic, N= nuclear, r= Spearman correlation coefficient

Correlation is significant at the 0.01 level (2-tailed), Bold denotes"Positive association"

Table S4 Associations between Jagged-1 and Notch-3 Protein in ISCC patients

Proteins Expression			Notch-3			JAG1		
			C	N	C+N	C	N	C+N
Jagged-1	C	r	**0.479**	**0.379**	**0.484**	1.000	**0.877**	**0.952**
		p value	**0.0001**	**0.0001**	**0.0001**	-	**0.0001**	**0.0001**
	N	r	**0.479**	**0.379**	**0.484**	**0.877**	1.000	**0.961**
		p value	**0.0001**	**0.0001**	**0.0001**	**0.0001**	-	**0.0001**
	C+N	r	**0.407**	**0.404**	**0.453**	**0.952**	**0.968**	1.000
		p value	**0.0001**	**0.0001**	**0.0001**	**0.0001**	**0.0001**	-
Notch-3	C	r	1.000	**0.600**	**0.907**	**0.479**	**0.407**	**0.463**
		p value	-	**0.0001**	**0.0001**	**0.0001**	**0.0001**	**0.0001**
	N	r	**0.600**	1.000	**0.840**	**0.379**	**0.404**	**0.412**
		p value	**0.0001**	-	**0.0001**	**0.0001**	**0.0001**	**0.0001**
	C+N	r	**0.907**	**0.840**	1.000	**0.484**	**0.453**	**0.493**
		p value	**0.0001**	**0.0001**	-	**0.0001**	**0.0001**	**0.0001**

Abbreviations: C= cytoplasmic, N= nuclear, r= Spearman correlation coefficient

Correlation is significant at the 0.01 level (2-tailed), Bold denotes “Positive association”

Figure S1 Uncropped western blots used in Figure 1g

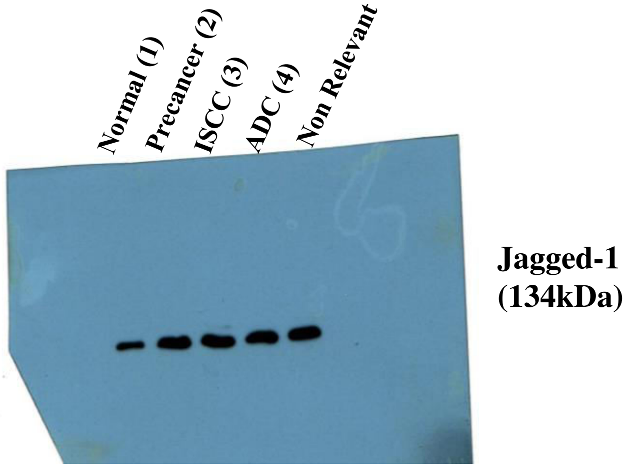

a.

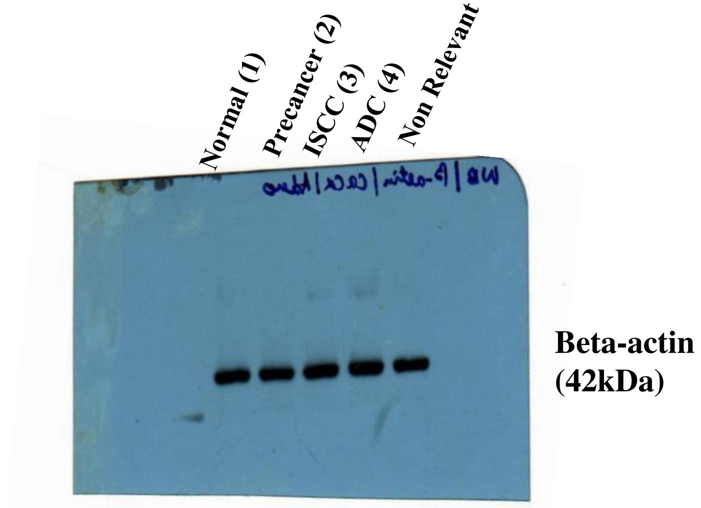

b.

Figure 1g

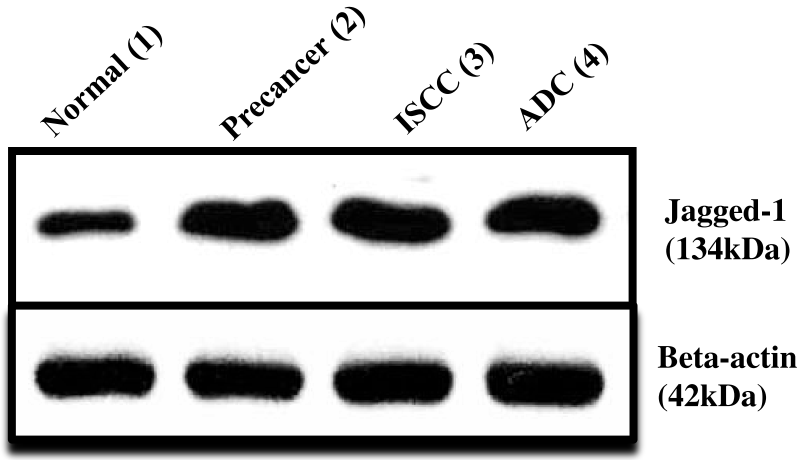

Figure S2 Uncropped western blot used in Figure 1h

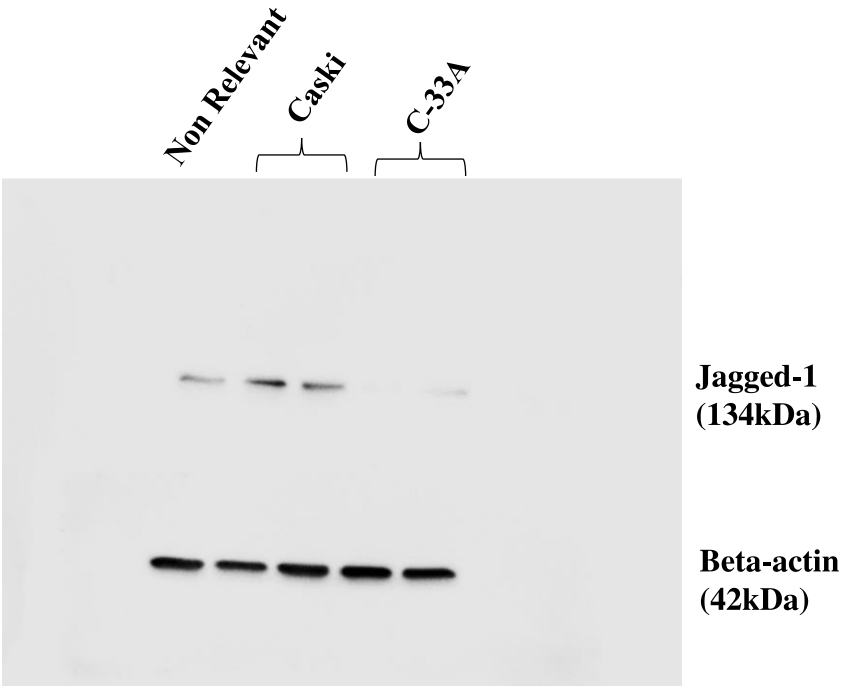

Figure 1h

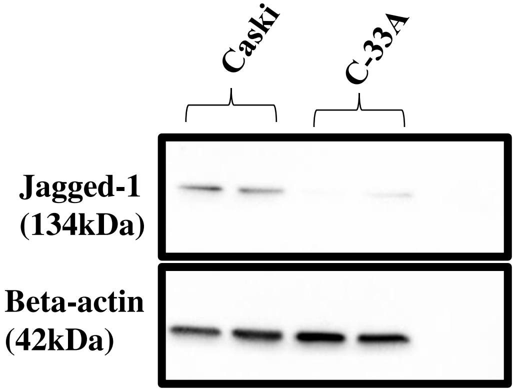

Supplement: Supplementary file 1 — Supplement S1, Table-S1, Table-S2,Table-S3, Table-S4, Figure S1, Figure S2 [file 41598_2018_27699_MOESM1_ESM.pdf]
